# Prospects of micromass culture technology in tissue engineering

**DOI:** 10.1186/1746-160X-3-4

**Published:** 2007-01-09

**Authors:** Jörg GK Handschel, Rita A Depprich, Norbert R Kübler, Hans-Peter Wiesmann, Michelle Ommerborn, Ulrich Meyer

**Affiliations:** 1 Department for Cranio- and Maxillofacial Surgery, Heinrich-Heine-University Düsseldorf, Moorenstr. 5, 40225 Düsseldorf, Germany; 2 Department for Cranio- and Maxillofacial Surgery, Westfälische-Wilhelms-Universität Münster, Waldeyerstr. 30, 48149 Münster, Germany; 3 Department for Operative and Preventive Dentistry and Endodontics, Heinrich-Heine-University Düsseldorf, Moorenstr. 5, 40225 Düsseldorf, Germany

## Abstract

Tissue engineering of bone and cartilage tissue for subsequent implantation is of growing interest in cranio- and maxillofacial surgery. Commonly it is performed by using cells coaxed with scaffolds. Recently, there is a controversy concerning the use of artificial scaffolds compared to the use of a natural matrix. Therefore, new approaches called micromass technology have been invented to overcome these problems by avoiding the need for scaffolds. Technically, cells are dissociated and the dispersed cells are then reaggregated into cellular spheres. The micromass technology approach enables investigators to follow tissue formation from single cell sources to organised spheres in a controlled environment. Thus, the inherent fundamentals of tissue engineering are better revealed. Additionally, as the newly formed tissue is devoid of an artificial material, it resembles more closely the *in vivo *situation. The purpose of this review is to provide an insight into the fundamentals and the technique of micromass cell culture used to study bone tissue engineering.

## Background

The *in vitro *formation of bone- or cartilaginous-like tissue for subsequent implantation [1-3] is, as described, commonly performed by using scaffolds. Recently, there is a controversy (e.g. biocompatibility, biodegradability) concerning the use of artificial scaffolds compared to the use of a natural matrix [4]. Skeletal defect regeneration by extracorporally created tissues commonly exploits a three-dimensional cell-containing artificial scaffold. As indicated before, a number of *in vitro *studies have been performed to evaluate the cell behaviour in various three-dimensional artificial scaffold materials [5-7]. Whereas most of these materials were generally shown to allow spacing of skeletal cells in a three-dimensional space, not all materials promote the ingrowth of cells within the scaffolds [8]. Rather, supporting cellular function depends, as described, on multiple parameters such as the chosen cell line, the underlying material, the surface properties and the scaffold structure. Some *in vitro *studies indicate that a material itself may impair the outcome of *ex vivo *tissue formation, when compared to a natural tissue-containing matrix. Additionally, in the *in vivo *situation defect regeneration can be critically impaired by the immunogenity of the material, the unpredictable degradation time and by side effects caused by degradation products [4]. Based on these consideration matrices close to the natural extracellular matrix are regarded as most promising in skeletal tissue engineering by some researchers. A recently elaborated approach in extracorporal tissue engineering is therefore the avoidance of non-degradable scaffolds, that are resorbed at a different time rate than the skeletal tissue regeneration by itself proceeds. Therefore, new approaches have been invented to overcome these problems by renouncing scaffolds.

## What is the theory of micromass technique?

It is well known that tissue explants can regenerate complete organisms [9]. Basic research has indicated that regeneration of simple animals and microtissues can be achieved by re-aggregation approaches using micromass technique [10]. Investigations on skeletal development gave first insight into this micromass biology [11-13]. The micromass technology relies to a great extent on the presence of the proteinacious extracellular matrix. As described before, the extracellular matrix may exert both direct and indirect influences on cells and consequently modulate their behaviour. At the same time, these cells alter the composition of the extracellular matrix. This may be accomplished in a variety of fashions, including differential expression of particular extracellular matrix components and/or proteases such as metalloproteinases by cells in the local microenvironment. Whereas most investigations concerning micromass technology were performed in developmental studies, only limited literature is available concerning the use of this technique in tissue engineering [14]. A large body of evidence has confirmed that a minimal cell number is required in three-dimensional tissue-like constructs to induce the differentiation of mesenchymal precursors along the chondrogenic and osteogenic pathways (reviewed in [15]). In contrast, mesenchymal precursors seeded in low-density micromasses adopt features of a fibroblastic phenotype and abolish cell differentiation, when mimicking a low-density condensation [16-18]. These findings indicated that a "critical" cell mass is necessary to proceed with a specific extracellular matrix formation. A threshold amount of precursor cells is necessary to form a three dimensional extracellular matrix structure around these cell masses promoting their differentiation. The extracellular matrix in the microenvironment then interacts with cells to further develop towards a specific tissue. The absence of the requisite extracellular matrix components would lead to decreased recruitment of precursors to the condensations, causing a subsequent deficiency in chondrocyte or osteoblast differentiation. *In vitro *studies with chondrocytes confirmed these findings, showing that the ability of mesenchymal precursors to initiate chondrogenic differentiation is dependent upon cell configuration within a condensation process, which varies by the density of the condensation [19].

## Technical aspects of the micromass technology

In the context of tissue engineering, *ex vivo *tissue generation may be optimised by the use of cell re-aggregation technology. The re-aggregate approach is a method to generate, in an attempt to mimic the *in vivo *situation, a tissue-like construct from dispersed cells, under special culture conditions. Therefore, the self-renewal (cell amplification), spatial sorting and self-organisation of multipotential stem cells in combination with the self-assembly of determined cells are the basis for such an engineering design option. Technically, cells are dissociated and the dispersed cells are then reaggregated into cellular spheres [14]. In order to technically refine scaffold-free spheres, cells are kept either in regular culture dishes (as gravitory cultures), in spinner flasks, or in more sophisticated bioreactors. In contrast to conventional monolayer cell cultures, in which cells grow in only two dimensions on the flat surface of a plastic dish, suspension cultures allow tissue growth in all three dimensions. It was observed that cells in spheres exert higher proliferation rates than cells in monolayer cultures, and their differentiation more closely resembles that seen *in situ*. This finding may be based on the spatial configuration in a three-dimensional matrix network. Different culture parameters (sizes of the culture plate, movement in a bioreactor, coating of culture walls) are all crucial to the process. Roller tube culture system have been shown to be suitable for cultivation of tissue explants in suspension. The cultivated and fabricated tissues may be used for studying the primary mixing of cells, and the patterns of cell differentiation and growth within growing spheres in order to improve the outcome of microsphere cultivation. In addition, some culture conditions could aid the development of high-throughput systems, and allow manipulation of individual spheres. It seems worthwhile elaborating new bioreactor technologies and culture techniques to improve the *ex vivo *growth of scaffold-free tissues. Technically, short-term re-aggregation experiments, which last from minutes to a few hours, can be distinguished from long-term studies. Short-term re-aggregation has been used widely to evaluate basic principles of cell-cell interactions and cell-matrix interactions, whereas long-term cultivation (days to several weeks) is suitable in *ex vivo *tissue engineering strategies. Recent studies on the re-aggregation approach aim to solve two aspects: to fabricate scaffold-free, three-dimensional tissue formation and at the same time to investigate basic principles of cellular self-assembly [20,21]. As in monolayer cultures, which facilitates the study of cell-material interactions, suspension cultures allow the evaluation of cell action towards a three-dimensional space. The re-aggregate approach enables to follow tissue formation from single cell sources to organised spheres in a controlled environment. Thus, the inherent fundamentals of tissue engineering are better revealed. Additionally, as the newly formed tissue is avoid of an artificial material, it more closely resembles the *in vivo *situation.

## Cell sources for micromass technology

Cells from cartilage and/or bone were found to be a suitable cell source for such *ex vivo *re-aggregate approaches. Anderer and Libera [1] developed an autologous spheroid system to culture chondrocytes and osteoblasts without adding xenogenous serum, growth factors, or scaffolds, considering that several growth factors and scaffolds are not permitted for use in clinical applications. It was demonstrated by such an approach that autologous chondrocytes and osteoblasts cultured in the presence of autologous serum form a three-dimensional micro-tissue that had generated its own extracellular matrix. Chondrocyte-based micro-tissue had a characteristic extracellular space that was similar to the natural matrix of hyaline cartilage. Osteoblasts were also able to build up a micro-tissue similar to that of bone repair tissue without collagen-associated mineral formation. The fabrication of a self-assembled skeletal tissue seems not to be limited towards certain species, as results from bovine and porcine chondrocyte and osteoblast cultivation led to the formation of species-related cartilage-like or bone-like tissue. However, conditions allowing cartilage formation in one species are not necessarily transposable to other species. Therefore, results with animal models should be cautiously applied to humans. In addition, for tissue-engineering purposes, the number of cell duplications must be, for each species, carefully monitored to remain in the range of amplification allowing redifferentiation and chondrogenesis [22].

It was recently observed, that even complex cellular systems can be generated *ex vivo *without the use of scaffolds. Co-cultures of osteoblasts and endothelial cells for example resulted in the formation of a bi-cellular micromass tissue renouncing any other materials. Other organotypic cultures, used to develop engineered tissues other then of skeletal origin, confirm that it is feasible to create tissue substitutes based on re-aggregated spheres technology. Examples of these strategies include liver reconstruction, synthesis of an artificial pancreas, restoration of heart valve tissue and cardiac organogenesis *in vitro *[23].

## Future prospects and challenges

Several investigations have suggested that after *in vivo *transfer of such reaggregates, tissue healing is improved in sense of a repair tissue that mimics the features of the original skeletal tissue [1,24]. Especially preclinical and clinical cartilage repair studies demonstrated that tissue formation resembled more closely the natural situation. The transplantation of reassembled chondrogenic micro-tissues is able to impair the formation of fibro-cartilage by suppression of type I collagen expression, while promoting the formation of proteoglycan accompanied by a distinct expression of type II collagen. It can be assumed that the volume of the observed repair tissue was formed by the implanted chondrospheres itself as well as by host cells located in the superficial cartilage defect. The mechanisms by which chondrospheres promote defect healing are complex and not completely understood. Van der Kraan et al. [4] reviewed the role of the extracellular matrix in the regulation of chondrocyte function in the defect site and the relevance for cartilage tissue engineering. Numerous other studies have confirmed that extracellular matrix of articular cartilage can be maintained by a distinct number of chondrocytes and that the extracellular matrix plays an important role in the regulation of chondrocyte function. In *in vitro*-generated cartilage-like tissue a time-dependent increase in the expression of collagen type II, S-100, and cartilage-specific proteoglycans, paralleled by a reduced cell-matrix ratio was observed in the microspheres [24]. The transplanted cell/matrix complex was attributed to be responsible for the observed chondrocyte proliferation, differentiation and hyaline cartilage-like matrix maturation *in vivo*.

The inductive properties of the implantation site may also be beneficial when a stem cell-based micro-tissue strategy is chosen. Stem cell tissue engineering using fetal or adult stem cells in combination with sphere technologies leads to implantable stem cell-driven tissues (unpublished data). Typically, stem cells must be amplified to large quantities in suspension cultures and have access to appropriate growth factors to establish specially organised histotypical spheres. These spheres can then be implanted into the lesioned skeletal site. Although adult stem cells of various origins can transdifferentiate into distinct cell types, the transformation of these cell types into functioning tissues and their successful implantation by re-aggregation technology needs further elaboration.
